# Angiopoietin-2 impairs collateral artery growth associated with the suppression of the infiltration of macrophages in mouse hindlimb ischaemia

**DOI:** 10.1186/s12967-016-1055-x

**Published:** 2016-10-26

**Authors:** Xiaoyong Tan, Kai Yan, Meiping Ren, Ni Chen, Yongjie Li, Xin Deng, Liqun Wang, Rong Li, Mao Luo, Yong Liu, Yan Liu, Jianbo Wu

**Affiliations:** 1 Drug Discovery Research Center, Southwest Medical University, Luzhou, Sichuan China; 2 Laboratory for Cardiovascular Pharmacology of Department of Pharmacology, The School of Pharmacy, Southwest Medical University, Luzhou, Sichuan China; 3 Renshou People’s Hospital, Renshou, Sichuan China; 4 Department of Internal Medicine, University of Missouri School of Medicine, Columbia, MO USA

**Keywords:** Angiopoietin-2, Ischaemia, Arteriogenesis, Macrophage

## Abstract

**Background:**

Angiopoietin-2 (Ang-2), a ligand of the Tie-2 receptor, plays an important role in maintaining endothelial cells and in destabilizing blood vessels. Collateral artery growth (arteriogenesis) is a key adaptive response to arterial occlusion. It is unknown whether the destabilization of blood vessels by Ang-2 can affect arteriogenesis and modulate mononuclear cell function. This study aimed to investigate the effects of Ang-2 on collateral artery growth.

**Methods:**

Hindlimb ischaemia model was produced in C57BL/6 mice by femoral artery ligation. Blood flow perfusion was measured using a laser Doppler perfusion imager quantitative RT-PCR analysis was applied to identify the level of angiogenic factors.

**Results:**

After the induction of hindlimb ischaemia, blood flow recovery was impaired in mice treated with recombinant Ang-2 protein; this was accompanied by a reduction of peri-collateral macrophage infiltration. In addition, quantitative RT-PCR analysis revealed that Ang-2 treatment decreased monocyte chemotactic protein-1 (MCP-1), platelet-derived growth factor-BB (PDGF-BB) mRNA levels in ischaemic adductor muscles. Ang-2 can lead to macrophage M1/M2 polarization shift inhibition in the ischaemic muscles. Furthermore, Ang-2 reduced the in vitro inflammatory response in macrophages and vascular cells involved in arteriogenesis.

**Conclusions:**

Our results demonstrate that Ang-2 is essential for efficient arteriogenesis, which controls macrophage infiltration.

**Electronic supplementary material:**

The online version of this article (doi:10.1186/s12967-016-1055-x) contains supplementary material, which is available to authorized users.

## Background

Neovascularization is a complex process that involves vasculogenesis, angiogenesis, and arteriogenesis [1–3]. The principal stimuli for blood perfusion include tissue ischaemia and hypoxia, inflammation, and vascular shear stress. Angiogenesis is the growth of new blood vessels from pre-existing blood vessels, whereas arteriogenesis is the enlargement of existing blood vessels to form collaterals and requires the recruitment of pro-inflammatory cells. A number of local and circulating angiogenic factors are involved in arteriogenesis, including vascular endothelial growth factor (VEGF), basic fibroblast growth factor (bFGF), and angiopoietins. Previous studies have demonstrated that angiopoietin-2 (Ang-2) regulates both angiogenesis and arteriogenesis [4], with the concentration of Ang-2 being an important determinant of which effect is observed. However, the role of Ang-2 in neovascularization in vivo remains controversial [5–7] and needs to be elucidated. Given that published studies have predominantly used transgenic mice to compare the effects of normal versus loss of Ang-2 expression on collateral remodelling, relatively little is known about the impact of enhanced Ang-2 expression on this process.

There have been conflicting reports concerning the role of Ang-2 in postnatal neovascularization. For example, Ang-2 was shown to increase the length, but not the density, of neovessels in a corneal implant model [8]. However, in a model of hindlimb ischaemia, collateral formation was impaired in Ang-2 transgenic mice [6]. Moreover, still others have reported that the selective inhibition of Ang-2 activity promotes blood reperfusion following ischaemia through the recruitment of inflammatory cells and combination with VEGF [5, 9]. Finally, Ang-2 was shown to increase vascular destabilization and regression by inhibiting pericyte recruitment and Tie-2–mediated phosphorylation of focal adhesion kinase [10–12]. However, no studies have implicated Ang-2 in controlling signalling pathways that initiate ischemia-mediated vessel stabilization and blood flow perfusion.

Therefore, we hypothesized that Ang-2 plays an important role in the regulation of blood flow recovery during ischaemia through the modulation of collateral artery growth. In this study, we studied the effects of r-Ang-2 on blood flow perfusion and the density of pre-existing collateral vessels in a murine hindlimb ischaemia model. We also studied post-ischaemic alterations in gene expression and macrophage recruitment to examine the potential mechanisms by which r-Ang-2 regulates arteriogenesis.

## Methods

### Mouse hindlimb ischaemia model

Male C57BL/6 mice were anaesthetized with sodium pentobarbital (60 mg/kg body weight, intraperitoneally) or isoflurane (5 % by inhalation) and administered a single subcutaneous dose of buprenorphine hydrochloride (0.1 mg/kg) for analgesia. Additional sodium pentobarbital (12 mg/kg body weight) or 5 % isoflurane was given as needed to maintain anaesthesia. Mice were euthanized by cervical dislocation at the end of the experiment while still anaesthetized. Mice were subjected to unilateral hindlimb ischaemia as previously described [13]. Recombinant human Ang-2 (50 µg/mL) or vehicle (PBS) was injected intraperitoneally every other day beginning 1 day before surgery. Real-time PCR was performed on mouse hindlimb muscles. In some experiments, mouse hindlimbs were also used for immunostaining. All protocols for animal use were reviewed and approved by the Animal Care Committee of Southwest Medical University in accordance with Institutional Animal Care and Use Committee guidelines.

### Laser Doppler perfusion images

The ratio of blood flow in the ischaemic (left) limb to the normoxic (right) limb was measured using a laser Doppler perfusion imager (LDPI) (Moor Instruments, Devon, UK) as previously described [14, 15]. Non-invasive perfusion imaging of the adductor thigh region and the plantar foot of both limbs was performed under 1.125 % isoflurane/O_2_ anaesthesia at 37 ± 0.5 °C, before, immediately after, and at 1, 3, and 7 days following femoral artery ligation. Regions of interest were drawn to facilitate the analysis of specific anatomic regions. To minimize data variability caused by ambient light and temperature, the LDPI index was expressed as the ratio of left limb (ischaemic) to right limb (normoxic) blood flow.

### Assessment of ischaemic muscles histology

Mice were euthanized 7 days after surgery. The ischaemic gastrocnemius muscles were excised, embedded in paraffin or TISSUE TEK OCT compound, and stored at −80 °C. Cross-sections were prepared for immunohistochemical analysis. Capillary density was determined by immunostaining with anti-PECAM-1 antibody. The number of PECAM-1+ vessels was quantified in 5 microscopic fields in each of 3 cross-sections from each tissue specimen using ImagePro Plus software. Adductor muscle cryostat sections were obtained, and incubated with primary antibodies anti-Mac-3 (1:500, Pharmingen) and α-SMA (1:100, Santa Cruz Biotech). The secondary antibodies used were goat ant-rat IgG AlexaFluor 488-conjugated antibody and goat ant-rabbit IgG AlexaFluor 568-conjugated antibody (Molecular Probes, Invitrogen). Images were captured by a fluorescence microscopy (Leica).

In some experiments, cross-sections were stained with hematoxylin-eosin (HE). Photomicrographs were taken using a microscope (Leica), the collateral diameters were measured in 5 randomly selected fields per section. The mean value of these measurements was taken as a single point for each animal.

### Quantitative real-time PCR

Total RNA was extracted from hindlimb muscles using TRIzol (Invitrogen). RNA was pre-treated with deoxyribonuclease I (Invitrogen Life Technologies), and a SuperScript kit (Invitrogen Life Technologies) was used to synthesize cDNA according to the manufacturer’s recommendations. Each sample was analysed in duplicate with ribosomal 18S RNA used as an internal control. All fold changes in gene expression were determined using the 2^−ΔΔCT^ method. The values are presented as the mean ± SEM. All primers are listed in Additional file 1: Table S1.

### Migration assay

Macrophages were obtained by flushing the peritoneal cavity with phosphate-buffered saline (PBS) 4 days after intraperitoneal injection with 1 mL of a 3 % thioglycollate solution (Difco Laboratories, Sparks, MD). Macrophage migration assays were performed using transwell migration chambers with 8.0-μm porous membranes (Corning Costar, Corning, NY, USA). Cells (2 × 10^4^) were added to the upper chambers and were treated with r-Ang-2 (200 nmol/L) or vehicle control. In some experiments, cells were pre-treated with r-Ang-2 for 30 min, followed by stimulation with MCP-1 (100 ng/mL). After incubation for 24 h at 37 °C in a humidified chamber with 5 % CO_2_, the porous membranes were rinsed, and the cells remaining in the upper chamber were removed with a cotton swab. The membranes were then fixed and stained with 0.5 % crystal violet. The cells that had migrated to the lower chamber were counted.

The migration of human coronary artery smooth muscle cells (VSMCs) was measured using a previously described method [16]. Briefly, cells were grown to confluence on 6-well plates. Parallel lines were then drawn with a marker on the back of each well, and linear wounds were made perpendicular to these lines in the cell monolayers by scraping each well with a sterile 200-µL pipette tip. Cells were then rinsed twice with serum-free medium to remove cellular debris. Images of the intersections between the parallel lines and wounds were obtained using an Olympus inverted microscope. The cells were then incubated with or without Ang-2 (200 ng/mL) for 30 min in the presence or absence of PDGF-BB (20 ng/mL). After 12 h, images of each intersection were obtained again. ImageJ software was then used to measure the distance between the two edges of each wound. The data are presented as the moving distance, which is the difference in the distances between the two edges at the same crossing at 0 and 12 h.

### Enzyme-linked immunosorbent assay

A human Ang-2 ELISA kit (R&D Systems) was used to quantify Ang-2 according to the manufacturer’s instructions.

### Statistical analysis

The data are expressed as the mean ± SEM of at least triplicate experiments. Groups were compared using the two-tailed Student’s *t* test.

## Results

### r-Ang-2 impaired blood reperfusion following ischaemia

Hindlimb ischaemia was induced in C57BL/6 mice by ligation and excision of the femoral artery, after which mice received either r-Ang-2 or vehicle control.

**Fig. 1:**
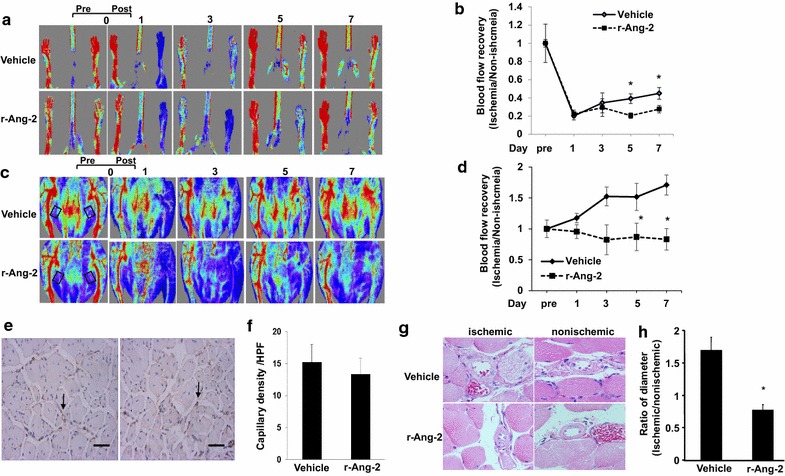
r-Ang-2 impaired blood flow perfusion during ischaemia. **a** Representative laser-Doppler images of the plantar foot at the indicated time points after femoral artery ligation. “Pre” and “Post” are immediately before and after surgery, respectively. **b** The mean blood flow ratio of ischaemic to non-ischaemic hindlimb foot pads for all animals at indicated time points (n = 6/group; *P < 0.05 vs. the r-Ang-2 groupat 5 and 7 days). **c** Representative laser-Doppler images of the adductor region at the indicated time points after femoral artery ligation. “Pre” and “Post” are immediately before and after surgery, respectively.* Outlined* areas shown the regions of interest. **d** The mean blood flow ratio in ischaemic to non-ischaemic hindlimb footpads for all animals at the indicated time points (n = 6/group; *P < 0.05 vs. r-Ang-2 group at 5 and 7 days). **e** Representative images of PECAM-1+ capillaries in ischaemic gastrocnemius muscles 7 days after femoral artery ligation. *Scale bars* 100 μm. **f** Mean capillary density in ischaemic gastrocnemius muscles assessed by PECAM-1 immunostaining. **g** Representative images of collateral arterioles in ischaemic adductor muscles, assessed using H&E staining. **h** The ratio of mean arteriole diameter in Ang-2 group to vehicle in ischaemic adductor muscles (n = 6/group; *P < 0.05 vs. vehicle)

To analyse circulating levels of Ang-2, mice were injected with recombinant protein, and plasma was collected after 7 days of treatment, and analyzed by ELISA. Intraperitoneal injections of r-Ang-2 showed that human Ang-2 (81.9 ± 8.08 ng/mL) was detectable in the plasma. r-Ang-2 was not detectable in both nonischemic and ischemic adductor muscles (data not shown). When we measured overall hindlimb blood flow, there were no significant differences in plantar foot perfusion between r-Ang-2- and vehicle control-treated mice (Fig. 1a, b). In addition, the PECAM-1+ capillary density in ischaemic gastrocnemius muscles did not differ significantly between the experimental groups (Fig. 1e, f).We next measured blood perfusion in the adductor region, which contains pre-existing collateral arterioles that supply the lower limb. Laser Doppler imaging revealed that reperfusion of ischaemic hindlimb tissue after femoral artery ligation was significantly reduced in r-Ang-2-treated mice compared with vehicle controls (Fig. 1c, d). Consistent with these results, the ratio of mean arteriole diameter in Ang-2 group to vehicle in ischaemic adductor muscles of r-Ang-2-treated mice 7 days after induction of ischaemia was significantly smaller than in vehicle control-treated animals (Fig. 1g, h).

### r-Ang-2 suppresses the infiltration of inflammatory cells

**Fig. 2:**
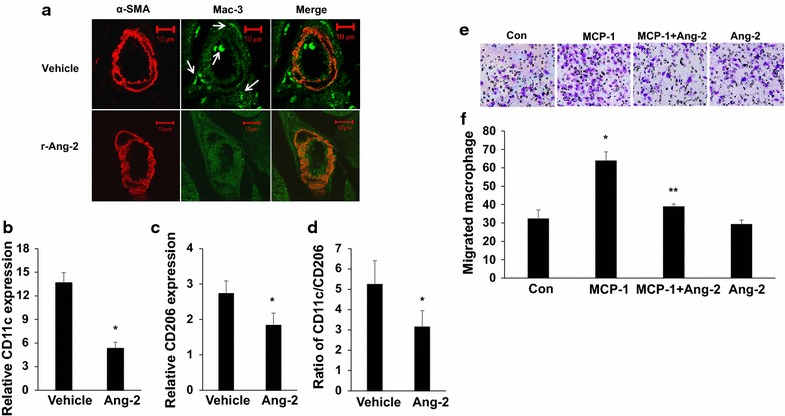
r-Ang-2 suppresses inflammatory cell infiltration. **a** Recruitment of macrophages in response to ischaemia as determined by Mac-3 staining. Representative images of macrophages surrounding collateral arterioles in ischaemic adductor muscles in r-Ang-2 treated-mice on day 7 after femoral artery ligation are shown. **b** mRNA expression of CD11c (M1 marker), and **c** mRNA expression of CD206 (M2 marker) in ischemic adductor muscles as measured by real-time PCR. **d** Ratio of CD11c to CD206, The data were normalized to the expression level of 18S rRNA in each sample. All values represent the mean ± SEM (n = 6/group). *P < 0.05 vs. vehicle control. **e** Thioglycollate-elicited mouse peritoneal macrophages were used to evaluate macrophage migration. Cells were added to the upper chambers of cell culture inserts and incubated with Ang-2 (200 ng/mL) or vehicle control and then treated with MCP-1 (100 ng/mL) or vehicle control as indicated. Representative images of macrophages that migrated to lower-chamber side of transwell membrane. **f** The data represent the mean of triplicate experiments. *P < 0.05 vs. negative control (cells exposed neither MCP-1 nor Ang-2). **P < 0.05 vs. macrophages incubated with MCP-1 only

To determine whether r-Ang-2 treatment could be related to inflammatory cell infiltration in ischaemia, we examined the infiltration of macrophages in ischaemic adductor muscles. The infiltration of macrophages around sites of arteriogenesis in ischaemic hind limbs was determined by immunostaining with the anti-Mac3 antibody. Treatment with r-Ang-2 caused a marked reduction in the infiltration of macrophages surrounding collateral arterioles in ischaemic adductor muscles 7 days post-femoral ligation (Fig. 2a). We next performed quantitative RT-PCR to study the effects of r-Ang-2 on macrophage polarization of ischemic muscles. Macrophage phenotype was characterized by examining expression of specific pro-inflammatory M1 (CD11c) and anti-inflammatory M2 (CD206) macrophage markers using real-time PCR. After 7 days of ischemia, both CD11c mRNA and CD206 mRNA expression were significantly downregulated in ischemic adductor muscles from r-Ang-2-treated mice compared with vehicle-treated mice (5.33 ± 0.78 vs. 13.66 ± 1.31 for CD11c; 1.83 ± 0.34 vs. 2.73 ± 0.36 for CD206; respectively, P < 0.05 for all) (Fig. 2b, c).The ratio of CD11c to CD206 (index of M1/M2 macrophage balance) was significantly lower in r-Ang-2-treated mice than n vehicle-treated mice (3.15 ± 0.79 vs. 5.24 ± 1.17; P < 0.05) (Fig. 2d).

To further study the effects of r-Ang-2 on macrophage migration, we isolated thioglycollate-elicited macrophages from C57BL/6 mice and used a modified Boyden chamber assay that required macrophages to migrate through a porous membrane. In the absence of adding Ang-2, MCP-1 significantly stimulated macrophage migration. However, pre-treatment with r-Ang-2 abrogated MCP-1-induced migration, whereas treatment with Ang-2 alone did not significantly alter macrophage migration (Fig. 2e, f).These results supported the functional significance of r-Ang-2 by inhibiting pericollateral macrophage recruitment.

### r-Ang-2 modulates post-ischaemic gene regulation

**Fig. 3:**
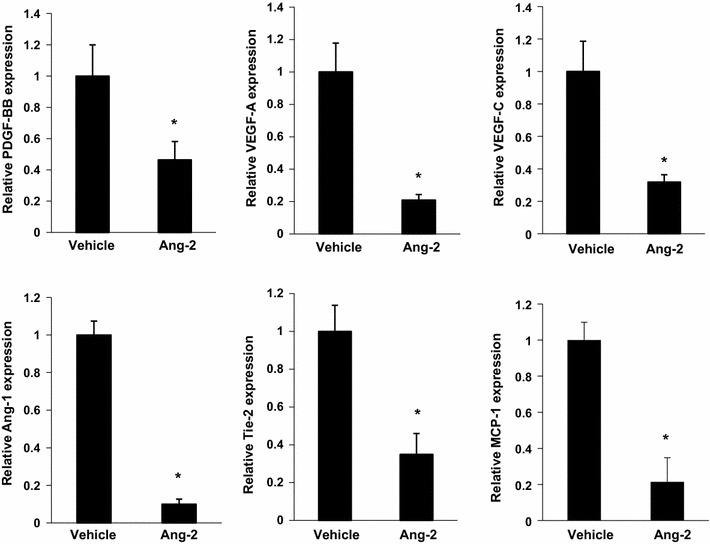
r-Ang-2 modulates post-ischaemic gene regulation. mRNA expression levels of angiopoietin-1, Tie-2, PDGF-BB, VEGF-A, VEGF-C, and MCP-1 were analysed using real-time PCR in ischaemic adductor muscles. The data were normalized to the expression level of 18S rRNA in each sample. All values represent the mean ± SEM (n = 6/group). *P < 0.05 vs. vehicle control

H&E staining showed reduced mean arteriole diameter in the adductor muscles from the r-Ang-2-treated group compared to the vehicle control group. To study differential gene expression after r-Ang-2 treatment, total RNA isolated from the adductor muscle was used to assess the gene expression levels of angiogenesis-related factors and their receptors. Quantitative real-time RT-PCR analysis revealed that the expression of PDGF-BB, VEGF-A and VEGF -C, angiopoietin-1 (Ang-1), and its receptor, Tie-2, was downregulated in the adductor muscles of r-Ang-2-treated mice (1.00 ± 0.19 vs. 0.46 ± 0.12 for PDGF-BB; 1.00 ± 0.18 vs. 0.21 ± 0.03 for VEGF-A; 1.00 ± 0.19 vs. 0.32 ± 0.05 for VEGF-C; 1.00 ± 0.07 vs. 0.09 ± 0.02 for angiopoietin-1; 1.00 ± 0.14 vs. 0.34 ± 0.11 for Tie-2; respectively, P < 0.05 for all) (Fig. 3). Additionally, MCP-1 has been shown to be downregulated after r-Ang-2 treatment and is known to be involved in the process of collateral formation (1.00 ± 0.08 vs. 0.19 ± 0.03; P < 0.05) (Fig. 3). These results suggest that angiogenesis is severely impaired in response to increased Ang-2 levels.

### Effects of r-Ang-2 on in vitro VSMC migration

**Fig. 4:**
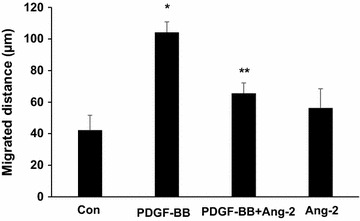
Effects of r-Ang-2 on VSMC migration in vitro. Migration of human coronary artery smooth muscle cells in the presence of r-Ang-2, followed by treatment with PDGF-BB (20 ng/mL) or vehicle control was studied. The data represent the mean of triplicate experiments. *P < 0.05 vs. negative control. **P < 0.05 vs. cells incubated with PDGF-BB only

Because we observed a decreased arteriole diameter in ischaemic adductor muscles in response to in vivo Ang-2 treatment, we studied the effect of Ang-2 on VSMCs migration in vitro using a 2-dimensional scratch assay. To determine if Ang-2 exhibits differential effects on VSMCs, we treated cultured human coronary artery smooth muscle cells with PDGF-BB. PDGF-BB significantly induced VSMCs migration, with maximal increases observed after 12 h of exposure. However, pre-treatment with Ang-2 attenuated the capacity of PDGF-BB to induce VSMC migration (Fig. 4). Furthermore, in the absence of added PDGF-BB, Ang-2 had no significant effects on migration (Fig. 4). These results further support the assertion that Ang-2 inhibits the recruitment of VSMCs.

## Discussion

In this study, we assessed the role of Ang-2 in arteriogenesis using a mouse hindlimb ischaemia model. Our findings indicate that Ang-2 reduced the diameter of collateral arterioles in ischaemic adductor muscles following ligation of the femoral artery. This change is associated with reductions in arteriogenesis but leaves angiogenesis intact. Furthermore, we found that Ang-2 reduced MCP-1-induced macrophage migration and PDGF-BB-induced VSMC migration in vitro. Therefore, we conclude that Ang-2 is important for arteriogenesis induced by acute loss of hindlimb perfusion.

The angiopoietin/Tie system is essential for vascular development and maturation [17]. Ang-1/Tie-2 signalling promotes blood vessel stabilization and the recruitment of mural cells, whereas Ang-2 acts as an Ang-1 antagonist and destabilizes vessels [18, 19]. However, the complex role of Ang-2 in vascular remodelling still remains unknown. We found that treatment with r-Ang-2 impaired the recovery of blood flow in the ischaemic hindlimb, suggesting that Ang-2 is involved in collateral artery growth after arterial ligation. However, effects of Ang-2 in hindlimb ischaemia remain controversial. For example, Tressel et al. showed that the Ang-2 inhibitor, L1-10, induced in ischemic tissue plays a critical role in blood flow recovery by stimulating inflammation and arteriogenesis [5]. However, Reiss et al. found that transgenic overexpression of Ang-2 impaired revascularization during hindlimb ischaemia. The importance of local variations in Ang-2 concentration might be an important determinant of whether vessel growth or regression occurs [6]. Our findings with r-Ang-2 in this study are in agreement with another murine study of Ang-2 overexpression and support the in vivo relevance of Ang-2-mediated destabilization of collateral arteries. We did not observe an increase in capillary density in ischaemic gastrocnemius muscle in response to r-Ang-2 treatment. In addition, our finding that the expression of several major growth factors was transiently downregulated following Ang-2 treatment further supports the initial role of Ang-2 in neovascularization. However, Ang-2 exhibits an opposing role regulating angiogenesis through its receptor Tie-2 and integrin signalling [12]. Thus, these data suggest that Ang-2 mediates blood flow recovery mainly through arteriogenesis without affecting angiogenesis.

The reduced diameter of collateral arterioles in ischaemic adductor muscles in mice treated with r-Ang-2 could be the result of inhibited mural cell recruitment. However, it is unclear whether Ang-2 has a direct effect on the recruitment of VSMCs and pericytes [20]. A recent study showed that Ang-2 induced pericyte apoptosis via α3β1 integrin signalling in a model of diabetic retinopathy [21]. In the present study, we further evaluated the effect of Ang-2 on VSMC migration. In the absence of exogenous Ang-2, PDGF-BB significantly enhanced the migration of VSMCs. However, in the absence of exogenous PDGF-BB, Ang-2 did not significant impact migration. The inhibitory effect of Ang-2 could be mediated by the uncoupling of PDGF receptor-β (PDGFR-β)-integrin crosstalk, as has been described [20, 22]. This mechanism was not resolved in our experiments. Additional studies are necessary to more precisely define the molecular events underlying Ang-2-mediated, integrin-dependent downregulation of PDGFR-β signalling.

There is evidence that Ang-2 acts as a chemoattractant for regulating monocyte/macrophage infiltration [5, 23], suggesting that treatment with r-Ang-2 may increase macrophage recruitment to remodelling collateral arterioles. However, our data did not support this mechanism because we observed reduced macrophage content in ischaemic adductor muscles in vivo. Moreover, Ang-2 treatment abrogated MCP-1-induced macrophage migration in vitro, whereas treatment with Ang-2 alone had no significant effects on migration. In the present study demonstrate that Ang-2 can lead to M1/M2 shift inhibition in the ischemic muscles. Our data suggest that Ang-2-mediated reductions in MCP-1 may impair monocyte recruitment and, therefore, arteriogenesis.

A limitation to the present study is that we cannot at this time definitively determine the inhibitory effect of Ang-2 on arteriogenic activity depends on macrophages compared with pericytes. Additionally, we only detected the effect of Ang-2 at this early time point. Future studies are required in which monocyte/macrophage populations are depleted and late adaptive responses to hind limb ischaemia assessed. In addition, cross-breeding of Apo E knockout mice with Ang-2 knockout or Ang-2 transgenic mice will provide additional evidence for the role of Ang-2 in atherogenesis. Despite these limitations, we believe our study provides a preliminary results of Ang-2 involved in the complex processes contributing to collateral formation.

## Conclusions

We found that Ang-2-treated mice exhibited impaired collateral remodelling after femoral artery ligation, as well as a reduction in the diameter of pre-existing collaterals. Ang-2 treatment also altered the expression of several growth factor genes and reduced pro-arteriogenic inflammatory responses. These findings indicate that Ang-2 could be a key regulator of post-ischaemic blood flow recovery by inhibiting the inflammatory factors required for arteriogenesis, and this may lead to potential therapeutic strategies for managing ischaemic cardiovascular diseases.

## Additional file

**Additional file 1: Table S1.** Sequences of primers used in the study.

## Abbreviations

Ang-2: angiopoietin-2
ApoE: apolipoprotein E
MCP-1: monocyte chemotactic protein-1
PDGF-BB: platelet-derived growth factor BB
PDGFR-β: platelet-derived growth factor receptor-β
r-Ang-2: recombinant angiopoietin-2
VEGF-A: vascular endothelial growth factor-A
VEGF-C: vascular endothelial growth factor-C
VSMC: vascular smooth muscle cell
