# Supplementary material for: Angiopoietin-2 impairs collateral artery growth associated with the suppression of the infiltration of macrophages in mouse hindlimb ischaemia
Source: J Transl Med. 2016 Oct 26;14:306. doi: 10.1186/s12967-016-1055-x (PMC5080762; doi:10.1186/s12967-016-1055-x)
Supplement: Supplementary file 1 — Additional file 1: Table S1. Sequences of primers used in the study. [file 12967_2016_1055_MOESM1_ESM.docx]

**Supplemental Table S1 Sequences of primers used in the study**

| Mmu-Ang-1 | Forward：CTTCCAGAACACGACGGGAA  Reverse：TGTATCTGGGCCATCTCCGA |
| --- | --- |
| Mmu-Tie2 | Forward：TGTATCTGGGCCATCTCCGA  Reverse：CCAACAGTGATGTCTGGTCCTATG |
| Mmu-VEGF-A | Forward：GCAGGCTGCTGTAACGATGAA  Reverse：TCACATCTGCTGTGCTGTAGGA |
| Mmu-VEGF-C | Forward：CCGGTGCATGTCTAAACTGGAT  Reverse：GCATCGGCACATGTAGTTATTCC |
| Mmu-PDGF-BB | Forward：CAGTGACCTTGGAGGACCAC  Reverse：GAATGGTCACCCGAGCTTGA |
| Mmu-MCP-1 | Forward：CAGCCAGATGCAGTTAACGC  Reverse：GCCTACTCATTGGGATCATCTTG |
| Mmu-CD11c | Forward：ACACAGTGTGCTCCAGTATGA  Reverse：GCCCAGGGATATGTTCACAGC |
| Mmu-CD206 | Forward：CATGGATGTTGATGGCTACTGGAG  Reverse：GTCTGTTCTGACTCTGGACACTTG |
| Mmu-18S | Forward：CCTGGATACCGCAGCTAGGA  Reverse：GCGGCGCAATACGAATGCCCC |
